# Association between brominated flame retardants and obesity: a mediation analysis through markers of oxidative stress and inflammation

**DOI:** 10.1265/ehpm.24-00328

**Published:** 2025-05-10

**Authors:** Yue Fei, Yulan Cheng, Xiangdong Wang, Jialing Ruan, Dongnan Zheng, Haotian Cao, Xuehai Wang, Xiaoke Wang, Xinyuan Zhao, Jinxian Yang

**Affiliations:** 1 Department of Occupational Medicine and Environmental Toxicology, Nantong Key Laboratory of Environmental Toxicology, School of Public Health, Nantong University, Nantong 226019, China; 2 Xinglin College, Nantong University, Qidong, Jiangsu, 226236, China

**Keywords:** NHANES, Brominated flame retardants, Obesity, Weighted quantile sum regression, Bayesian kernel machine regression

## Abstract

**Background:**

Recent studies have provided compelling evidence that exposure to brominated flame retardants (BFRs) can adversely affect human health. We aim to explore the potential impact of BFRs on adiposity and central obesity.

**Methods:**

Data from the National Health and Nutrition Examination Surveys (NHANES) cycles conducted between 2009 and 2014 was used to study the connections between variables. After filtering, we analyzed a sample of 4,110 adults aged 20 years and above. Our goal was to examine the potential association between BFRs and consequences and investigate the part played by oxidative stress and inflammatory markers as intermediaries. To achieve this, we used advanced statistical methods such as weighted quantile sum (WQS) regression, quantile-based g-computation (QGC), and the Bayesian kernel machine regression (BKMR).

**Results:**

The findings showed that among the examined chemicals, exposure to PBDE85 (weight: 41%), PBDE100 (24%), and PBB153 (23%) may be the dominant contributors to general obesity risk. Upon controlling for all variables that could impact the results, it was found that the QGC outcomes indicated a positive correlation between exposure to mixtures of brominated flame retardants and the occurrence of abdominal obesity (OR = 1.187, 95% CI: 1.056–1.334, p = 0.004). Significant contributions were made by PBDE85 (52%), PBB153 (27%), and PBDE100 (21%). Mediation analysis shows that lymphatic cells (LC) and albumin (ALB) partially mediate the link between brominated flame retardants and obesity. The results of BKMR are generally consistent with those of WQS and QGC.

**Conclusion:**

At a population level, our research has revealed a noteworthy correlation between BFRs and obesity. However, further investigation is required through prospective cohort studies and in-depth mechanistic exploratory studies.

**Supplementary information:**

The online version contains supplementary material available at https://doi.org/10.1265/ehpm.24-00328.

## 1. Introduction

Flame retardants (FRs) are a diverse class of chemical compounds widely used to delay or inhibit the spread of fires. However, many FRs have been found to have detrimental effects on the environment and human health [1]. Organic halogen flame retardants represented by brominated flame retardants (BFRs) have had an extensive range of uses around the world for many years due to their excellent flame-retardant effect, such as additives for synthetics, fabrics, furniture, automobiles, electrical and electronic appliances [2, 3]. Brominated flame retardants (BFRs) are a group of chemicals with diverse physiochemical properties employed in various industrial applications to reduce flammability, which include polybrominated diphenyl ethers (PBDEs), polybrominated biphenyls (PBBs), and other brominated compounds [4]. Certain manufactured substances found in the surroundings can dissolve in fat and accumulate in fatty tissue [5]. BFRs are among the most common endocrine-disrupting environmental chemicals in this category [6]. Since most BFRs are highly fat soluble, bioaccumulation of toxins in animal fat and biomagnification in the food chain can lead to their accumulation in relatively high concentrations in the human body [3]. The risk of metabolic dysfunction in adipose tissue could be increased by BFRs, according to an in vitro cellular study. This elevated risk could significantly impact the liver’s metabolism [7]. Indoor dust and intake of seafood and dairy products have been reported to be the main sources of exposure to BFRs for the general adult population, while breast milk is the main route of exposure for children, and in addition, recycling of electronic waste, etc. is an important route of exposure to BFRs for occupational personnel [4, 8]. A review has shown that BFRs have raised extensive concern during past decades for their long-term residue, bioaccumulation, semivolatile, and potential toxicity to the environment and human health [9]. Epidemiological studies have shown that exposure to BFRs may do harm to humankind health. For instance, it has been positively associated with some diseases such as thyroid disorders, developmental disorders, reproductive health, and cardiovascular disease [10–12]. At the same time, a recent study has shown that there is a significant positive association between human exposure to BFRs and the risk of metabolic syndrome (MetS) [3]. Moreover, recent animal experiments have shown that adult male C57BL/6 mice exposed to BDE-209 for 60 d had more significant liver and adipose tissue weights than the control group [13]. As described above, it is essential to research the disorders in lipid metabolism, such as obesity, caused by BFRs, with crucial points on solid evidence that remain poor.

Obesity and overweight are conditions characterized by the accumulation of excessive body fat, leading to various health complications [14, 15]. Globally, the prevalence of overweight and obesity continues to increase, with obese individuals making up approximately 30% of the world’s population, totaling over 2 billion people [16]. According to the Centers for Disease Control and Prevention, between 1999–2000 and March 2020, the rate of obesity in the United States witnessed a significant rise, from 30.5% to 41.9%. Similarly, the rate of severe obesity also saw an upward trend, increasing from 4.7% to 9.2% [17]. Previous research in 2015 has suggested that nearly 4.0 million global deaths are attributed to high body mass index (BMI). Cardiovascular disease accounted for over 66% of deaths linked to high BMI [18]. There is adequate evidence to suggest a correlation between obesity and type 2 diabetes, liver disease, and various types of cancer based on previous observational studies [19, 20]. Numerous studies have generated several innovative approaches to tackle the issue of obesity [21].

Research has demonstrated that BFRs can cause disruptions in the processing of glycolipids and trigger an inflammatory response during the initial phases of fat accumulation and throughout prolonged obesity, resulting in chronic activation of the immune system [22, 23]. Meanwhile, a study among young adults found that metabolically unhealthy obese patients had higher levels of oxidative stress parameters compared to normal weight and metabolically healthy patients [24]. Therefore, we hypothesized that exposure to BFRs may be associated with obesity in humans and that oxidative stress and inflammation are key factors in the mechanisms of BFR exposure and obesity development.

In summary, for the following reasons, we used the data from the National Health and Nutrition Examination Surveys (NHANES) database, which is sourced from the American population, to explore the correlation between BFRs exposure and the risk of obesity, as well as the possible mediating roles of inflammation and oxidative stress. Firstly, although the American market has recognized the toxicity of PBDEs and gradually phased out related products, due to the environmental persistence of PBDEs, exposure in the environment is still widespread [25]. Secondly, the occurrence of obesity is prevalent worldwide and data from the U.S. CDC also shows that the obesity rate among the population remains at a relatively high level [26]. Moreover, cross-sectional studies based on the American population have indicated that there is a correlation between exposure to environmental pollutants and the risk of obesity [27]. Finally, the NHANES database contains a large amount of comprehensive data on biological sample tests, questionnaires, and physical measurements of the American population, including the data required for our cross-sectional study.

## 2. Methods

### 2.1 Study population

This cross-sectional study accessed all data from the National Health and Nutrition Examination Survey (NHANES) database. NHANES is a cross-sectional study that aims to evaluate the health and nutritional status of adults and children in the United States. It is conducted annually by the National Center for Health Statistics (NCHS) and the Centers for Disease Control and Prevention (CDC) and recruits around 5000 participants yearly. The study is nationally representative and is crucial in informing public health policies and interventions. The sampling process used by NHANES is a sophisticated stratified multistage approach that incorporates data from various sources such as demographics, diet, physical examination, laboratory tests, and questionnaires. Our analyses investigated data from three consecutive NHANES cycles from 2009–2014. We have included 5,183 individuals who are older than 20 years of age in our study. All of them have undergone measurements of all serum BFRs. To ensure the accuracy of the results, individuals who met any one of the following criteria were excluded from the study: (1) those whose BMI and WC data was unavailable (n = 284); (2) those with missing covariates (n = 764); and (3) those without data available for mediator markers (n = 20). The analyses ultimately included 4110 adults (Fig. S1).

### 2.2 Measurement of serum BFRs and definition of obesity

The NHANES Laboratory Procedures Manual is a comprehensive guide that offers in-depth instructions on how to effectively collect, store, and process blood specimens [28]. Automated liquid-liquid extraction and sample clean-up were used to measure 11 polybrominated diphenyl ethers (PBDEs) and PBB-153 in serum, as per the NHANES dataset. In the research analysis, we focused solely on PBB-153 and eight PBDEs that exhibited a detection rate of over 75% (Table S1). The values that fell below the limit of detection (LOD) were replaced with the square root of two.

### 2.3 Outcome variables

Anthropometric measurements and biospecimens were collected in each mobile examination center (MEC). Individuals with a BMI of 30 kg/m^2^ or more were classified as obese, according to the World Health Organization reference [14]. In men, abdominal obesity is characterized by a waist circumference of 102 cm or more, while in women, it is characterized by a waist circumference of 88 cm or more [29].

### 2.4 Oxidative stress and chronic inflammation markers

In some cross-sectional studies of the American population based on the NHANES database, when it comes to inflammation and oxidative stress markers as mediators, the main oxidative stress markers include serum total bilirubin, iron levels, and albumin, while the inflammation markers include alkaline phosphatase, γ-glutamyl transferase (GGT), ferritin, neutrophil count, lymphocyte count, c-reactive protein and neutrophil-to-lymphocyte ratio [30–33]. In our study, due to the certain differences in data across different cycles, some inflammation markers were either absent or present in too small amounts during the study period. Therefore, the selection of inflammation and oxidative stress mediators in this study was based on previous NHANES database research using these biomarkers as mediators, referencing mediators used in NHANES studies with similar exposures, while considering specific circumstances of each survey cycle. The oxidative stress and inflammation markers finally included in our study were total bilirubin, albumin, and serum iron (as oxidative stress markers), as well as alkaline phosphatase (ALP), segmented neutrophil count, and lymphocyte count (as inflammation markers) [34, 35].

### 2.5 Covariates

We have considered various demographic and health-related factors to conduct an accurate analysis. These factors include age, gender, race, educational level, marital status, poverty income ratio, cotinine level, alcohol consumption, hypertension, and diabetes. Age is a categorical variable, while gender is either male or female. Race is categorized as Mexican American, Other Hispanic, Non-Hispanic White, Non-Hispanic Black, or Other Race. Educational level is classified as above high school, high school, or below high school. Marital status is divided into married/living with a partner, widowed/divorced/separated/never married. Poverty income ratio (PIR) is grouped as less than or equal to 1.3, 1.3–3.5, or higher than 3.5. Cotinine level is determined as either below LLOD or above LLOD, and alcohol consumption is either 12 drinks or fewer or more than 12 drinks. Lastly, the determination of hypertension and diabetes in the population was based on the respondent’s self-reported history of being told by a doctor or other health professional that they had hypertension or diabetes.

### 2.6 Statistical analysis

The descriptive analysis involved expressing continuous variables using the mean and standard deviation (SD), while categorical variables were represented by calculating frequencies and percentages. The Chi-square test was used to compare the percentages of categorical variables between the groups. At the same time, the T-test was employed to compare differences in continuous variables between the obesity and non-obesity groups. The model assumptions were met by conducting a log transformation of the concentration of serum BFR and six other intermediate indicator concentrations. We also conducted separate descriptive analyses for male and female gender. We used a heat map to display the correlation between the exposure pairs by calculating Spearman’s correlation coefficients for every combination of chemicals whose concentrations were transformed using the natural logarithm.

The NHANES study used a complicated and multi-level sampling method. To investigate the possible links between individual BFR and both overall obesity and abdominal obesity, we employed weighted multiple logistic regression models and adjusted for confounding variables. This allowed us to determine odds ratios (ORs) and their corresponding 95% confidence intervals (CIs). We analyzed logistic regression to explore the links between obesity and 9 BFR chemicals. The participants’ BFR concentrations were divided into four groups, with quartiles being utilized for this purpose. We calculated the odds ratios (ORs) and 95% confidence intervals (CIs) for the prevalence of general obesity or abdominal obesity in the second (Q2), third (Q3), and fourth (Q4) quartiles of serum BFR levels compared to the first (Q1) quartile. In binary logistic regression, a model that incorporated all confounders was used. We also explored the potential non-linear associations between individual BFR and general or abdominal obesity by restricted cubic spline (RCS).

Environmental pollutants typically have multiple effects on the human body rather than just one factor. We utilized the weighted quantile sum (WQS) regression and the Quantile g-computation (QGC) analysis to assess the global impact of BRF exposure on general and abdominal obesity. The WQS regression model has become popular due to its ability to evaluate the combined impact of the anticipated variables on the result [36]. WQS assumes that the exposures included are linear and additive and that their effect on the target outcome is unidirectional [37]. QGC can offer versatility in evaluating non-linear and opposing exposures and presenting the impact of each exposure in either a positive or negative manner [38]. We, therefore, used QGC analysis to evaluate multiple chemicals’ synergistic effects and screened for exposures that had a positive effect on outcome risk, which were then put into WQS for validation to satisfy model assumptions and exclude bias.

Based on existing cross-sectional studies in human populations, BFRs exposure is associated with an increased risk of non-alcoholic fatty liver disease and atherosclerosis, mediated by inflammation and oxidative stress, and is positively correlated with oxidative stress markers [34, 36, 39]. Therefore, we hypothesize that inflammation and oxidative stress may also mediate the relationship between BFRs exposure and the occurrence of obesity in our study, and we will conduct mediation analysis to verify the existence of such mediating effects. Two estimates need to be considered: Firstly, the average causal mediating effect (ACME) determines how exposure to a pollutant affects obesity risk through the mediating variable. Secondly, the average direct effect (ADE) determines how exposure affects the risk of obesity when the mediating variable is fixed at a certain level. In this study, the regression model of intermediary analysis is adjusted for all covariables.

### 2.7. Sensitivity analysis

Considering that traditional methods may be limited by multicollinearity and model selection errors, we introduce the Bayesian kernel machine regression (BKMR) to more accurately capture the complexity of mixing exposures. The impact of all the BFR metabolites in combination on the likelihood of general or abdominal obesity can be steadily assessed by BKMR [40]. The Markov chain Monte Carlo algorithm was used to conduct all BKMR models, with 20,000 iterations.

WQS, RCS, QGC, Mediation, and BKMR analyses were conducted via R software (version 4.2.2) by using “qgcomp,” “mediation,” “rms,” “gWQS” and “bkmr” packages, respectively. A two-tailed test was used to define the level of statistical significance as 0.05.

## 3. Results

### 3.1. Basic characteristics of participants

Table 1 revealed the essential characteristics of 4110 individuals, all 20 years or older, with 2042 (49.68%) males and 2068 (50.32%) females included in this study. The participants’ age, BMI, and WC mean values (with SD) were as follows: 48.97 (17.61) years, 29.06 (6.79) kg/m^2^, and 99.32 (16.37) cm, respectively. Participants were predominantly non-Hispanic whites, above high school, and more than 12 drinks. Therein, the prevalence of general obesity was 37.5%. Statistically, significant variations in the distributions of gender, age, race, education level, poverty income ratio, alcohol consumption, hypertension, and diabetes were discovered between the two groups. The prevalence of abdominal obesity was 56.8%, and the significant difference in distribution between the two groups was the same as in the case of general obesity. Table 2 demonstrates the geometric means and quartiles of serum BFRs and mediator markers. Moreover, Table S2 and Table S3 revealed that a higher general obesity prevalence in females than in males was observed (40.2% vs 34.7%), and women also have significantly higher rates of abdominal obesity than men (69.1% vs 40.3%). However, Table S4 shows that males have higher serum levels of BFRs than females. Notably, the inter-correlation between BFRs was weak to strong (rs range: 0.13–0.94). Amongst these, PBDE99 and PBDE85, along with PBDE47 and PBDE99, showed a high correlation (rs = 0.95, P < 0.001) (Fig. S2).

**Table 1:** Baseline characteristics of the study population, NHANES 2009–2014 (N = 4110).

**catalogs**	**General obesity**			**Abdominal obesity**		

	**No**	**Yes**	***P*-value**	**No**	**Yes**	***P*-value**
**Number of subjects (%)^a^**	2570 (62.5)	1540 (37.5)		1777 (43.2)	2333 (56.8)	
**Gender (%)^a^**			<0.001			<0.001
Male	1334 (65.3)	708 (34.7)		1138 (55.7)	904 (44.3)	
Female	1236 (59.8)	832 (40.2)		639 (30.9)	1429 (69.1)	
**Age (%)^a^**			0.001			<0.001
20–40 years	931 (65.8)	484 (34.2)		787 (55.6)	628 (44.4)	
40–60 years	802 (59.1)	555 (40.9)		546 (40.2)	811 (59.8)	
≥60 years	837 (62.6)	501 (37.4)		444 (33.2)	894 (66.8)	
**Race (%)^a^**			<0.001			<0.001
Mexican American	318 (56.2)	248 (43.8)		211 (37.3)	355 (62.7)	
Other Hispanic	250 (60.1)	166 (39.9)		180 (43.3)	236 (56.7)	
Non-Hispanic White	1173 (64.5)	647 (35.5)		744 (40.9)	1076 (59.1)	
Non-Hispanic Black	444 (52.3)	405 (47.7)		331 (39.0)	518 (61.0)	
Other race	385 (83.9)	74 (16.1)		311 (67.8)	148 (32.2)	
**Educational level (%)^a^**			<0.001			<0.001
Below high school	550 (58.4)	392 (41.6)		352 (37.4)	590 (62.6)	
High school	556 (60.0)	371 (40.0)		387 (41.7)	540 (58.3)	
Above high school	1464 (65.3)	777 (34.7)		1038 (46.3)	1203 (53.7)	
**Marital status (%)^a^**			0.261			0.559
Married/living with partner	1544 (63.3)	897 (36.7)		1065 (43.6)	1376 (56.4)	
Widowed/divorced/separated/never married	1026 (61.5)	643 (38.5)		712 (42.7)	957 (57.3)	
**Poverty income ratio (%)^a^**			0.002			0.004
≤1.3	813 (60.5)	531 (39.5)		547 (40.7)	797 (59.3)	
1.3–3.5	909 (60.9)	583 (39.1)		632 (42.4)	860 (57.6)	
>3.5	848 (66.6)	426 (33.4)		598 (46.9)	676 (63.1)	
**Body mass index (kg/m^2^), (mean (SD))^b^**	24.99 (3.05)	35.87 (5.79)	<0.001	24.15 (3.16)	32.81 (6.42)	<0.001
**Waist Circumference (cm), (mean (SD))^b^**	90.21 (10.09)	114.52 (13.27)	<0.001	86.46 (8.80)	109.11 (13.83)	<0.001
**Cotinine level (%)^a^**			0.43			0.096
Below LLOD	735 (63.5)	422 (36.5)		476 (41.1)	681 (58.9)	
Above LLOD	1835 (62.1)	1118 (37.9)		1301 (44.1)	1652 (55.9)	
**Alcohol consumption (%)^a^**			0.027			<0.001
12 drinks or fewer	644 (59.7)	435 (40.3)		389 (36.1)	690 (63.9)	
More than 12 drinks	1926 (63.5)	1105 (36.5)		1388 (45.8)	1643 (54.2)	
**Hypertension (%)^a^**	741 (50.0)	741 (50.0)	<0.001	416 (28.1)	1066 (71.9)	<0.001
**Diabetes (%)^a^**	208 (40.1)	311 (59.9)	<0.001	105 (20.2)	414 (79.8)	<0.001

^a^Number of participants and percentage. Chi-square test was used to compare the differences of categorical variables between participants with and without obesity.

^b^Mean value and standard deviation (SD). Student’s t-test was used to compare the differences of continuous variables between participants with and without obesity.

Note: Individuals with a body mass index (BMI) of 30 kg/m^2^ or more are classified as general obesity. Abdominal obesity is defined as a waist circumference of 102 centimeters or more for men and 88 centimeters or more for women.

**Table 2:** Geometric means and quartiles of serum BFRs and mediator markers. NHANES 2009–2014 (N = 4110).

**Catalogs**	**GM (95% CI)^a^**	**Median (IQR)^b^**
**Serum BFRs (pg/g)**		
PBB153	14.216 (13.749,14.702)	14.910 (6.615,27.100)
PBDE28	6.898 (6.787,7.015)	6.987 (4.717,10.010)
PBDE47	122.405 (120.181,124.711)	118.600 (81.620,183.525)
PBDE85	2.339 (2.289,2.389)	2.210 (1.484,3.708)
PBDE99	23.582 (23.058,24.119)	22.130 (14.600,37.260)
PBDE100	24.962 (24.484,25.432)	23.760 (16.060,36.940)
PBDE153	54.615 (53.464,55.757)	51.100 (34.260,82.960)
PBDE154	2.218 (2.173,2.266)	2.148 (1.419,3.414)
PBDE209	15.907 (15.627,16.184)	15.230 (10.920,20.960)
**Oxidative stress marker**		
Serum bilirubin (TBIL, umol/L)	11.151 (11.012,11.291)	11.970 (8.550,13.680)
Albumin (ALB, g/L)	42.447 (42.351,42.564)	43.000 (41.000,45.000)
Serum iron (SI, umol/L)	14.025 (13.846,14.211)	14.300 (11.100,18.600)
**Markers of chronic inflammation**		
Alkaline phosphatase (ALP, U/L)	64.477 (63.880,65.105)	64.000 (53.000,79.000)
Absolute neutrophil cell count (ANC, 1000 cells/uL)	3.906 (3.857,3.951)	3.900 (3.100,5.000)
Lymphocyte count (LC, 1000 cells/uL)	2.008 (1.988,2.028)	2.000 (1.600,2.500)

Note: PBB153: 2,2′,4,4′,5,5′-Hexabromobiphenyl; PBDE28: 2,4,4′-Tribromodiphenyl ether; PBDE47: 2,2′,4,4′-Tetrabromodiphenyl ether; PBDE85: 2,2′,3,4,4′-Tentabromodiphenyl ether; PBDE99: 2,2′,4,4′,5-Pentabromodiphenyl ether; PBDE100: 2,2′,4,4′,6-Pentabromodiphenyl ether; PBDE153: 2,2′,4,4′,5,5′-Hexabromodiphenyl ether; PBDE154: 2,2′,4,4′,5,6′-Hexabromodiphenyl ether; PBDE209: Decabromodiphenyl ether. ^a^G-Mean (95%). ^b^Median (25th, 75th percentiles).

### 3.2. Associations of individual BFRs with obesity

**Fig. 1:**
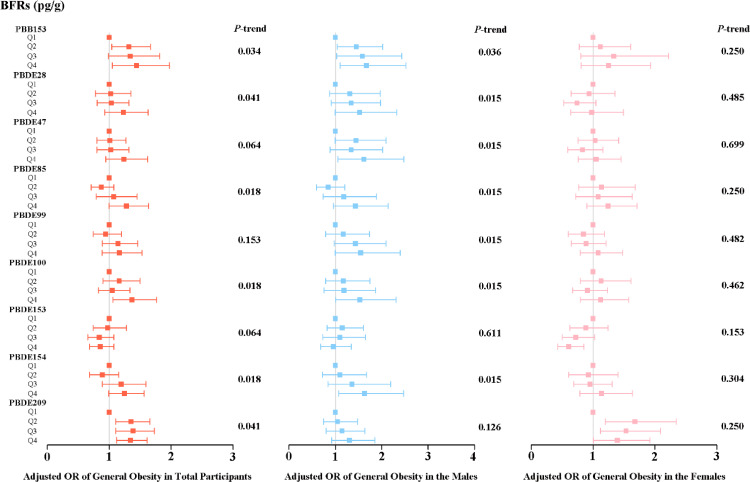
Weighted odds ratios (95% CIs) for general obesity by serum BFRs quartiles in participants. The squares and horizontal line represent for the ORs and 95% CIs. All of the models are adjusted for demographic characteristics (gender, age, race, educational levels, marital status and PIR), lifestyle (cotinine levels and alcohol consumption) and self-reported of hypertension and diabetes conditions.

**Fig. 2:**
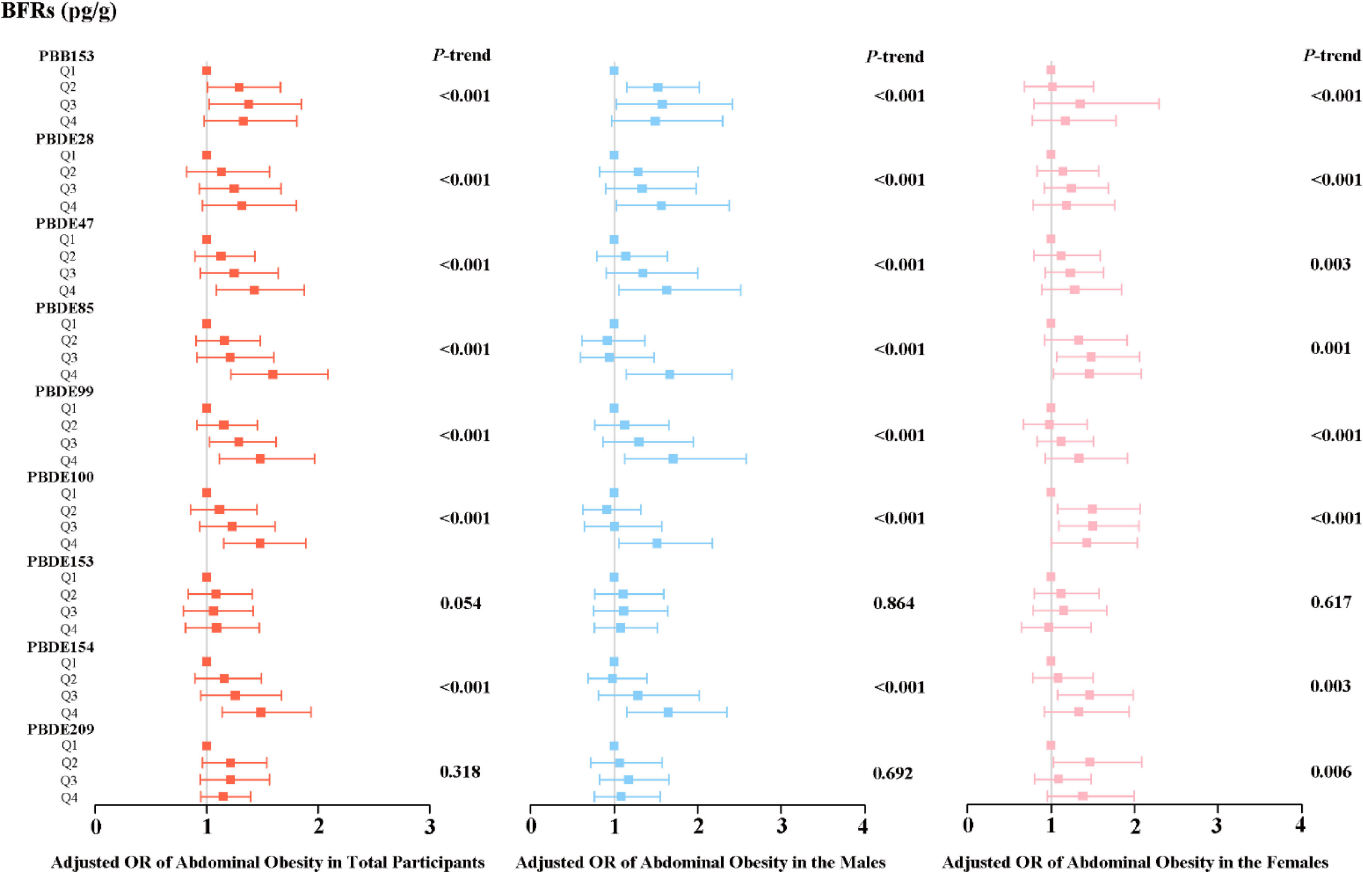
Weighted odds ratios (95% CIs) for abdominal obesity by serum BFRs quartiles in participants. The squares and horizontal line represent for the ORs and 95% CIs. All of the models are adjusted for demographic characteristics (gender, age, race, educational levels, marital status and PIR), lifestyle (cotinine levels and alcohol consumption) and self-reported of hypertension and diabetes conditions.

We utilized weighted multiple linear logistic regression models to examine the links between the prevalence of obesity and exposure to individual BFRs. After controlling the confounding variables, the three serum BFR variables (PBB153, PBDE100, PBDE209) remained significantly and positively associated with the incidence of general obesity. In detail, the risk of general obesity showed an increase in Q2 and Q4 when compared to Q1. The weighted odds ratio (OR) with 95% confidence intervals (CIs) for Q2 and Q4 were 1.317 (1.041–1.666) and 1.440 (1.052–1.973) respectively. However, only Q4 showed an increased risk of general obesity compared to Q1 for PBDE100, with an OR of 1.368 and a 95% CI of 1.060–1.765. Conversely, for PBDE209, the increased risk of general obesity was found in Q2 (OR = 1.352; 95% CI: 1.103–1.657), Q3 (OR = 1.382; 95% CI: 1.105–1.729), and Q4 (OR = 1.343; 95% CI: 1.117–1.614) when compared to Q1 (Fig. 1). When stratified by sex, associations were observed between serum BFRs and general obesity in men (PBB153; PBDE47; PBDE100; PBDE154). However, we did not observe meaningful results in females (Fig. 1). In addition, regarding abdominal obesity, the top quartile of PBDE47, PBDE85, PBDE99, PBDE100, and PBDE154 was found to have a higher likelihood of abdominal obesity compared to the lowest quartile. This association was represented by OR and 95% CI of 1.43 (1.09–1.88), 1.59 (1.22–2.09), 1.48 (1.11–1.97), 1.48 (1.16–1.89) and 1.48 (1.14–1.94), respectively, as shown in Fig. 2. When stratified by sex, associations were observed between serum BFRs and abdominal obesity in both men (PBDE28, PBDE47, PBDE85, PBDE99, PBDE100, PBDE154) and women (PBDE85, PBDE100) (Fig. 2).

A significant nonlinear relationship was found between general obesity and PBB153 (p-value for nonlinear = 0.022) and PBDE209 (p-value for nonlinear = 0.032), as shown in Fig. S3. Additionally, Fig. S4 illustrated that PBDE154 (p-value for nonlinear = 0.034) and PBDE209 (p-value for nonlinear = 0.040) demonstrated a considerable nonlinear correlation with abdominal obesity.

### 3.3. Association between co-exposure of serum BFRs with obesity

**Fig. 3:**
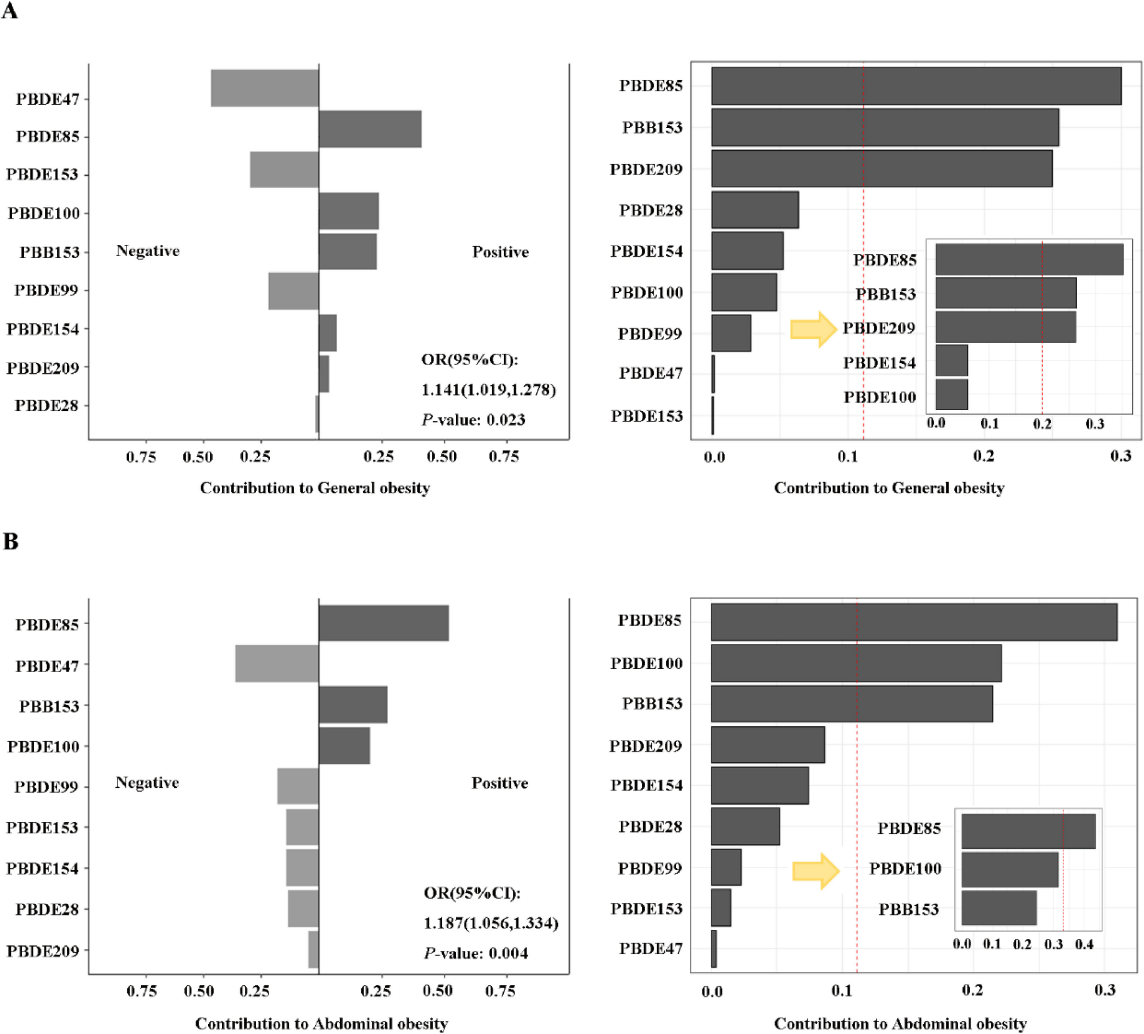
Association of serum BFRs mixture with obesity risk via QGC and WQS regression. The analysis was conducted using quantile-based g-computation (QGC) in the first step, followed by weighted quantile sum (WQS) regression in the second step after excluding exposure factors with negative weights. (A) Results of general obesity as an outcome. (B) Results of abdominal obesity as an outcome. All of the models are adjusted for demographic characteristics (gender, age, race, educational levels, marital status and PIR) and lifestyle (cotinine levels and alcohol consumption).

Using a single BFR is insufficient to determine the impact of exposure to a combination of BFRs on general and abdominal obesity. Therefore, we have employed the WQS regression and QGC models to examine how co-exposure might impact the levels of serum BFRs. The study conducted by QGC has found a positive correlation between an increase in exposure to a mixture of all chemicals examined and the risk of general obesity (OR = 1.141, 95% CI: 1.019–1.278, P < 0.05). Among the chemicals examined, PBDE85 (weight: 41%), PBDE100 (24%), and PBB153 (23%) may be the most significant contributors to general obesity risk. The findings obtained from QGC and WQS consistently indicate potential risk factors. After adjusting for all confounding factors, we analyzed the combined impact of exposure mixtures on abdominal obesity. The results showed that the QGC of mixture BFR exposure positively correlated with abdominal prevalence (OR = 1.187, 95% CI: 1.056–1.334, P = 0.004). Notably, PBDE85 (52%), PBB153 (27%), and PBDE100 (21%) had a significant impact, as shown in Fig. 3. The WQS regression results were consistent with the QGC findings, with PBB85 contributing the most. Both genders’ QGC and WQS results are displayed in Fig. S5.

### 3.4. Markers of inflammation and oxidative stress-mediated the association between BFRs and obesity

**Fig. 4:**
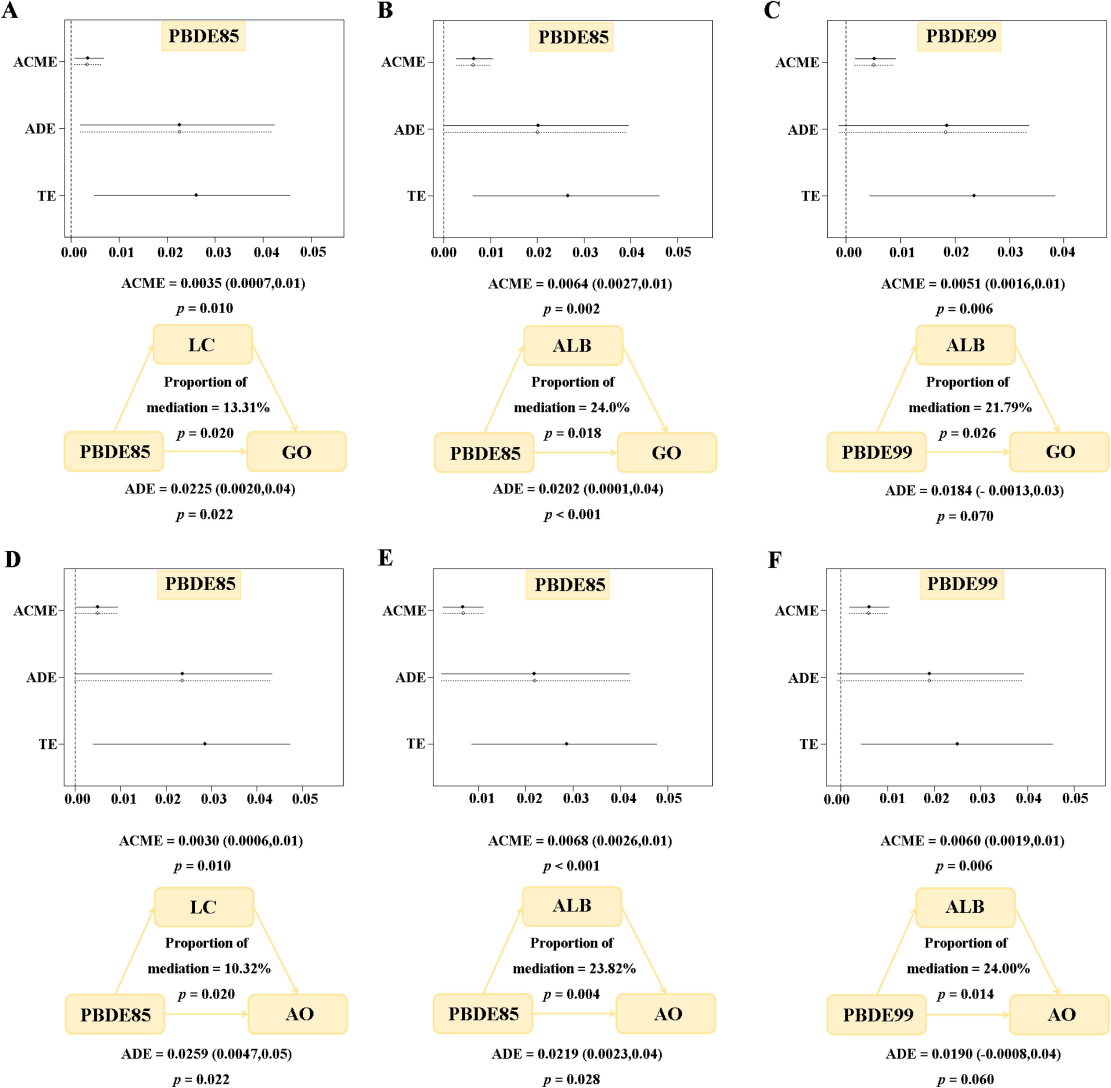
Mediating effect of oxidative stress and inflammation on BFRs exposure and obesity risk. Oxidative stress markers: (B), (C), (E) and (F) albumin (ALB). Inflammation markers: (A) and (D) lymphocyte count (LC). ACME: average causal mediation effect, ADE: average direct effect. All of the models are adjusted for demographic characteristics (gender, age, race, educational levels, marital status and PIR), lifestyle (cotinine levels and alcohol consumption) and self-reported of hypertension and diabetes conditions. Abbreviations: TE, Total Effect; GO, General Obesity; AO, Abdominal Obesity.

In this study, we conducted mediation analyses on all the proposed inflammation and oxidative stress markers, with each BFR and two obesity outcomes serving as the basis. The main statistically significant results we found are presented in Fig. 4. Figures 4A, 4B, and 4C show the mediating role of inflammatory and oxidative stress markers in the relationship between BFRs and general obesity. The three inflammatory and oxidative markers significantly mediated the association between PBDE85, PBDE99, and general obesity, with lymphatic cell (LC) and albumin (ALB) accounting for 13.31%, 24.0 and 21.79% of the association, respectively (P < 0.05). Meanwhile, we also explored the intermediary effects of inflammation and oxidative stress markers on abdominal obesity under exposure to BFRs. Figures 4D, 4E, 4F, LC, and ALB explained 10.32%, 23.82%, and 24.00% of the association, respectively.

### 3.5. Sensitivity analysis

The trend analysis using the BKMR model showed a notable increase in both general and abdominal obesity with increasing mixture concentrations. Figures S6A and S6D demonstrate that the trend was more noticeable when serum BFR concentrations were at their 60th to 75th percentile as compared to their 50th percentile. We can observe the univariate exposure-response functions (95% CI) between BFRs and the risk of general or abdominal obesity in Fig. S6C and Fig. S6F. These functions were obtained while maintaining the concentrations of the other eight serum BFRs at the median. Figure S7A and Fig. S7B reveal the Posterior inclusion probabilities (PIPs) of each BFR for general obesity or abdominal obesity in the total survey population.

## 4. Discussion

The associations were analyzed between BFRs and general obesity or abdominal obesity, which included 4110 US people aged 20 or older. Our research demonstrated that exposure to BFRs is linked to a higher risk of obesity. Specifically, we employed linear regression models to establish a connection between certain varieties of BFRs and obesity. Two distinct mixture analysis models, the WQS regression and the QGC models, were used to show a correlation between mixed exposure to BFRs and obesity. Furthermore, our mediation analysis indicated that particular inflammation and oxidative stress markers partially mediated the association between BFRs and obesity. Actually, inflammation and oxidative have been reported to be involved in most other diseases [41–43]. Moreover, the above two mechanisms also widely participated in pollutant-mediated toxicity [44–46].

Several scientists have also investigated the relationship between exposure to BFRs and adverse human health effects. A review indicates increasing evidence of a positive correlation between pollutants and human obesity [47]. A cross-sectional analysis of the US population indicated that individuals and a mixture of BFRs were positively associated with COPD [48]. Che et al. conducted a cross-sectional study and found that higher exposure to overall BFRs is associated with an increased risk of MetS and its components [3]. Meanwhile, Using the QGC model and WQS regression on NHANES data, Han et al. found a positive association between BFR exposure and markers of oxidative stress [36]. In addition, The NHANES study found that both individual and mixed organophosphate flame retardants (OPFRs) are linked to hyperuricemia, with inflammation playing a significant role [49]. However, further study is required to investigate the direct relationship between population exposure to BFRs and obesity. In line with our findings in large populations, in vitro and in vivo studies also suggest a possible association between exposure to brominated flame retardants and adipogenesis or weight gain. In vitro, Short- and long-term exposure of a pre-adipocyte (3T3-L1) cell line and a hepatocyte (HepG2) cell line to novel brominated flame retardants (NBFRs) has been found to increase the risk of metabolic dysfunction in adipose tissue and affect liver metabolism [7]. Studies conducted in vivo have demonstrated that male progeny exhibit adipocyte hypertrophy and an augmented rate of weight gain in the epididymal white adipose tissue (eWAT) under the influence of environmentally relevant doses of pentabromoethylbenzene (PBEB) when maternal mice are subjected to it [50].

On the one hand, in a study on occupational exposure to BFRs among chemical manufacturing workers, significant positive correlations were found between exposure levels of BFRs (particularly BDE-209) in biological samples and the levels of biomarkers such as total bilirubin, indirect bilirubin, and albumin/globulin [51]. An animal study on rats exposed to BFRs during gestation and early developmental stages found that serum alkaline phosphatase levels were altered following exposure [52]. On the other hand, in two separate in vivo experiments conducted on mice and zebrafish involving exposure to various BFRs, elevated levels of the oxidative stress marker malondialdehyde (MDA) were observed, inducing oxidative damage [53, 54]. In another animal study involving rats, exposure to BFRs was found to strongly induce the production of 8-hydroxy-2′-deoxyguanosine (8-OHdG), a biomarker of oxidative damage [55]. Regarding the correlation between inflammation and oxidative stress with obesity, studies have indicated that biomarkers of inflammation or oxidative stress are consistently associated with the risk of obesity and obesity-related diseases [56, 57]. These findings suggest that exposure to BFRs is likely to induce alterations in oxidative stress status and inflammatory levels, and that changes in these biomarker levels may play a pivotal role in the onset and progression of obesity [58, 59]. Based on the mediation analysis results of this study, it is plausible to hypothesize that oxidative stress and inflammation partially mediate the association between BFRs and population-level obesity, thereby offering mechanistic clues for subsequent investigations. At the same time, limited by the cross-sectional study design and data from the NHANES database, the research could not include more direct biomarkers such as MDA and 8-OHdG. In the future, we will further explore the impact of BFR exposure on obesity and clarify the roles of inflammation and oxidative stress by combining population-based findings with in vivo and in vitro experiments.

Certain BFRs, notably Polybrominated Diphenyl Ethers (PBDEs), have been demonstrated to accumulate in fat due to their lipophilicity [60]. Previous studies have shown that PBDEs can induce differentiation of 3T3-L1 cells into adipocytes by increasing IL-1β expression and decreasing PGC-1α and adiponectin expression [7, 61]. This study reveals the mediating role of inflammation-based markers and oxidative stress markers in the association between BFRs and the risk of obesity, which provides us with new ideas for exploring the mechanism. Firstly, an animal experiment found that PBDE may cause a decrease in 3-indolepropionic acid (3-IPA), which disrupts glucose and insulin signaling [62]. A substance called 3-IPA, which is a byproduct of tryptophan, is generated by bacteria in the intestines. This substance is strongly associated with the diet and has been demonstrated to prevent the synthesis of lipids in the liver and the production of inflammatory molecules [63]. An animal cohort experiment found that rats exposed to toxins and treated with IPA experienced a significant reduction in toxic-induced oxidative stress and pro-inflammatory responses. The experimental results show that co-treatment with IPA reversed chlorpyrifos (CPF) downregulation of Superoxide Dismutase (SOD), Glutathione Peroxidase (GPx), Glutathione S Transferase (GST) and (Reduced glutathione) GSH and decreased CPF upregulation of IL-1β (inflammation marker) [64]. Meanwhile, similar indoles can shield testes against lipid autoxidation and iron-triggered lipid peroxidation at therapeutic levels [65]. Secondly, a proposal put forward by a review is that obesity and inflammation are connected through gut microbes, suggesting that the lipopolysaccharide (LPS) produced by bacteria in the gut may lead to a subclinical inflammatory process and obesity [66]. A study conducted on animals showed that mice that received a constant subcutaneous injection of LPS over four weeks exhibited weight gain in their total body, liver, and adipose tissue similar to that of mice fed a high-fat diet [67]. So elevated plasma levels of LPS, due to increased intestinal permeability and a high-fat diet, contribute to the development of obesity and associated early inflammation [68]. Furthermore, in studies of Non-alcoholic fatty liver disease (NAFLD), there is evidence that LPS may be an inducer of oxidative stress activation in patients [69]. Given the unclear in vivo metabolism mechanism of BFRs, it is hypothesized that excessive exposure to BFRs may affect in vivo metabolism and lead to Intestinal disorders. Further work with experimental validation should be carried out to explore the underlying mechanism despite the evidence provided in this study.

The development of diseases often depends on the interaction and cooperation of mixed environmental pollutants. Thus, we focused on the combined effects of BFRs using the WQS, QGC, and BKMR models in addition to the univariate analyses. Previous studies have been limited by the traditional analytical strategy of modeling one chemical at a time or simply summing for all chemicals of interest. WQS regression and g-computation are combined in QGC to provide inferential simplicity and flexibility [37]. Given the potential for complex and nonlinear effects of exposures on health, we also utilized the BKMR model to identify the specific mixture components that have a negative impact on health. The analyses showed essentially consistent results across the models. The WQS models found that PBDE85 had the highest weight among all the chemicals, while the BKMR identified PBB153 as having the highest PIPs.

We observed gender differences in the distribution of BFRs in human serum, with higher serum levels of BFRs in males than in females. In vivo metabolism of PBDEs may produce stronger pseudoestrogens, as suggested by in vitro experiments [70]. Meanwhile, the antagonistic activity of NBFRs against the oestrogen receptor has been confirmed by in vitro experiments [71]. In addition, a study comparing the concentration of BFRs in the hair of men and women suggests that differences in exposure may be due to the amount of time spent in indoor environments [72]. These characteristics may account for gender differences, particularly in women.

We have a few significant advantages in our study. First, the NHANES offers a considerable and well-structured sample that is a good representation, guaranteeing that the results are valid and relevant to a larger audience. Second, three robust models, WQS, QGC, and BKMR, were used to investigate the independent and joint effects of BFR mixtures and their outcomes, with a focus on gender differences. Finally, this study also examines the impact of BFRs on two types of obesity, as well as their mechanisms of action in obesity, whose findings offer support for the creation of health protection measures against BFRs. While our research has various advantages, it also has some drawbacks that we must recognize. Initially, the current study’s cross-sectional design constrains our capacity to determine causality. Consequently, additional prospective research is required. Secondly, the analyzed data does not reflect recent human exposure to BFRs, as the NHANES database no longer collects data related to BFRs after 2016. Meanwhile, it is generally believed that the Reactive Oxygen Species (ROS) level is a well-recognized marker of oxidative stress, and some indicators (such as Tumor Necrosis Factor-alpha (TNF-α), Interleukin-6 (IL-6), etc.) are also commonly considered to be able to well reflect the body’s inflammation level [73]. However, limited by the types of data in the NHANES database, we can only select, based on previous studies, some widely-recognized inflammation markers (such as alkaline phosphatase and lymphocyte count) and oxidative stress markers (such as serum bilirubin and albumin) for which the NHANES database can provide data to include in our mediation analysis. Thirdly, even with multiple interpolations, lower BPF detection rates may still lead to uncertainty in composite exposure models. Finally, in our study, we controlled for many factors, but potential confounders still remained.

## 5. Conclusion

Our study concluded that there is a correlation between exposure to BFRs and obesity. The risk of obesity increases with higher exposure to overall BFRs. The significant chemicals identified for obesity are PBB153, PBDE100, and PBDE85. At the same time, our study highlights an association between BFRs and inflammation and oxidative stress markers, such as lymphocyte and albumin, which may explain BFRs link to obesity. Additional prospective cohort studies and in-depth mechanistic exploratory studies are required to comprehend the impact of BFRs on obesity and to investigate the causality and precise mechanisms involved. Our results could increase public knowledge about avoiding exposure to BFRs and encourage the exploration of safer alternatives that prioritize human health.

## Supplementary information

**Additional file 1:** **Fig. S1.** Flow diagram for selecting eligible participants from NHANES. **Fig. S2.** Pearson correlation coefficients between serum BFRs. **Fig. S3.** The continuous relationship of serum BFRs levels associated with general obesity based on RCS analysis. **Fig. S4.** The continuous relationship of serum BFRs levels associated with abdominal obesity based on RCS analysis. **Fig. S5.** Association between In-transformed serum BFRs mixture and the risk of general obesity and abdominal obesity in male and female participants, as assessed via quantile-based g-computation (first step) and weighted quantile sum regression (second step after exclusion of exposure factors with negative weights). **Fig. S6.** Association of obesity with BFRs estimated by Bayesian Kernel Machine Regression (BKMR) in participants. **Fig. S7.** Posterior inclusion probabilities (PIPs) of each BFR for outcome, using the Bayesian kernel machine regression (BKMR) model. **Table S1.** Detection rates of serum BFRs. NHANES 2009–2014 (N = 4110). **Table S2.** Population characteristics by general obesity and sex in adults, NHANES 2009–2014. **Table S3.** Population characteristics by abdominal obesity and sex in adults, NHANES 2009–2014. **Table S4.** Difference in arithmetic mean of serum BFRs between male and female. NHANES 2009–2014 (N = 4110).

## Declarations

### Availability of data and materials

Data will be made available on request.
