# Supplementary material for: Association between brominated flame retardants and obesity: a mediation analysis through markers of oxidative stress and inflammation
Source: Environ Health Prev Med. 2025 May 10;30:35. doi: 10.1265/ehpm.24-00328 (PMC12086098; doi:10.1265/ehpm.24-00328)
Supplement: Supplementary file 1 — Additional file 1: Fig. S1. Flow diagram for selecting eligible participants from NHANES. Fig. S2. Pearson correlation coefficients between serum BFRs. Fig. S3. The continuous relationship of serum BFRs levels associated with general obesity based on RCS analysis. Fig. S4. The continuous relationship of serum BFRs levels associated with abdominal obesity based on RCS analysis. Fig. S5. Association between In-transformed serum BFRs mixture and the risk of general obesity and abdominal obesity in male and female participants, as assessed via quantile-based g-computation (first step) and weighted quantile sum regression (second step after exclusion of exposure factors with negative weights). Fig. S6. Association of obesity with BFRs estimated by Bayesian Kernel Machine Regression (BKMR) in participants. Fig. S7. Posterior inclusion probabilities (PIPs) of each BFR for outcome, using the Bayesian kernel machine regression (BKMR) model. Table S1. Detection rates of serum BFRs. NHANES 2009–2014 (N = 4110). Table S2. Population characteristics by general obesity and sex in adults, NHANES 2009–2014. Table S3. Population characteristics by abdominal obesity and sex in adults, NHANES 2009–2014. Table S4. Difference in arithmetic mean of serum BFRs between male and female. NHANES 2009–2014 (N = 4110). [file ehpm-30-035-s001.docx]

**Supplementary material**

**Association between Brominated Flame Retardants and Obesity: A Mediation Analysis through Markers of Oxidative Stress and Inflammation**

Yue Fei ^1, #^, Yulan Cheng ^1, #^, Xiangdong Wang ^1^, Jialing Ruan ^1^, Dongnan Zheng ^1^, Haotian Cao ^1^, Xuehai Wang ^1^, Xiaoke Wang ^1, *^, Xinyuan Zhao ^1, *^, Jinxian Yang ^2, *^

^1^ Department of Occupational Medicine and Environmental Toxicology, Nantong Key Laboratory of Environmental Toxicology, School of Public Health, Nantong University, Nantong 226019, China

^2^ Xinglin College, Nantong University, Qidong, Jiangsu, 226236, China

^#^Contributed equally to this work.

*Correspondence to: Xinyuan Zhao, Email: [zhaoxinyuan@ntu.edu.cn](mailto:zhaoxinyuan@ntu.edu.cn); Xiaoke Wang, Email: [wxk11628@ntu.edu.cn;](mailto:liulei19920123@163.com;) or to Jinxian Yang, Email: [yjx0815@ntu.edu.cn](mailto:yjx0815@ntu.edu.cn).

**Fig. S1.** Flow diagram for selecting eligible participants from NHANES.

**Fig. S2.** Pearson correlation coefficients between serum BFRs.

**Fig. S3.** The continuous relationship of serum BFRs levels associated with general obesity based on RCS analysis.

**Fig. S4.** The continuous relationship of serum BFRs levels associated with abdominal obesity based on RCS analysis.

**Fig. S5.** Association between In-transformed serum BFRs mixture and the risk of general obesity and abdominal obesity in male and female participants, as assessed via quantile-based g-computation (first step) and weighted quantile sum regression (second step after exclusion of exposure factors with negative weights).

**Fig. S6.** Association of obesity with BFRs estimated by Bayesian Kernel Machine Regression (BKMR) in participants.

**Fig. S7.** Posterior inclusion probabilities (PIPs) of each BFR for outcome, using the Bayesian kernel machine regression (BKMR) model.

**Table S1.** Detection rates of serum BFRs. NHANES 2009-2014 (N = 4110).

**Table S2.** Population characteristics by general obesity and sex in adults, NHANES 2009 - 2014.

**Table S3.** Population characteristics by abdominal obesity and sex in adults, NHANES 2009 - 2014.

**Table S4.** Difference in arithmetic mean of serum BFRs between male and female. NHANES 2009-2014 (N = 4110).

Figure S1


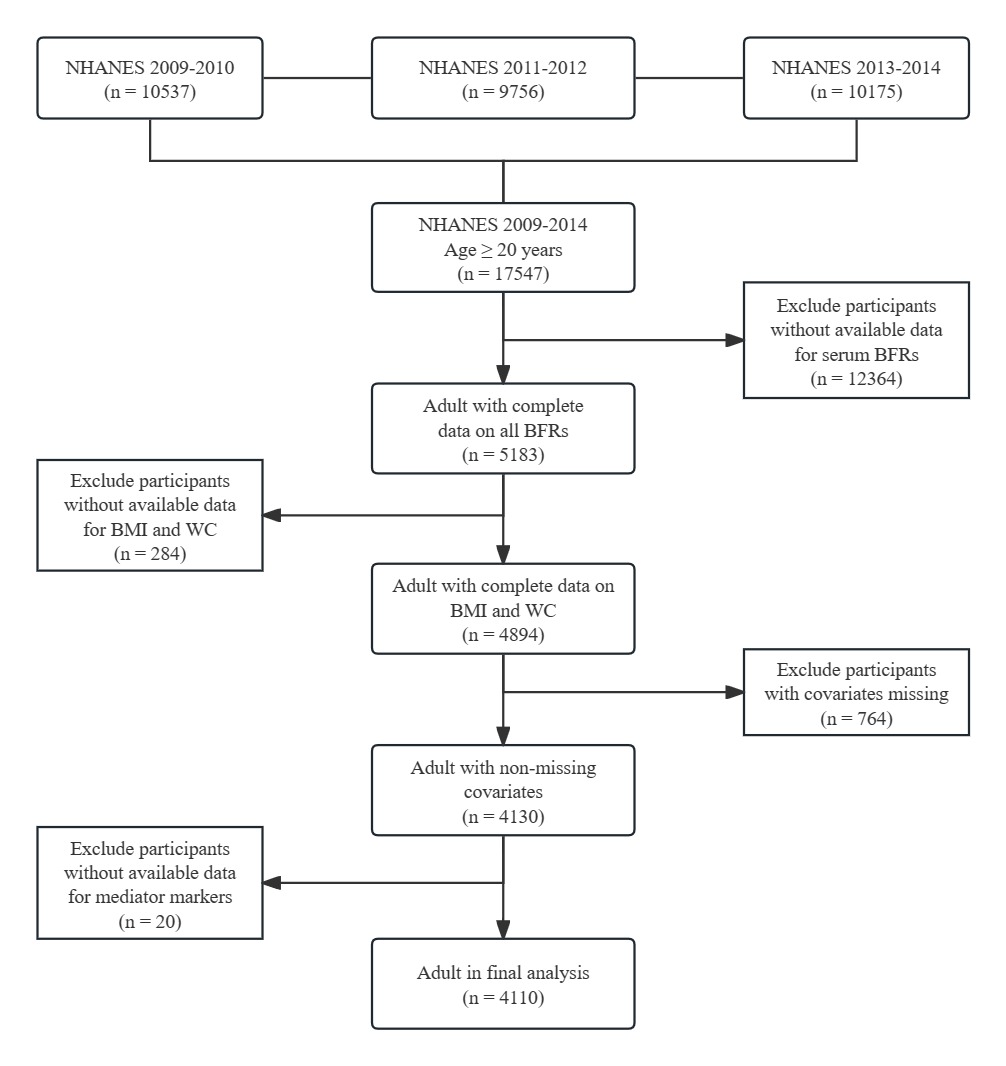


**Fig. S1. Flow diagram for selecting eligible participants from NHANES.**

Figure S2


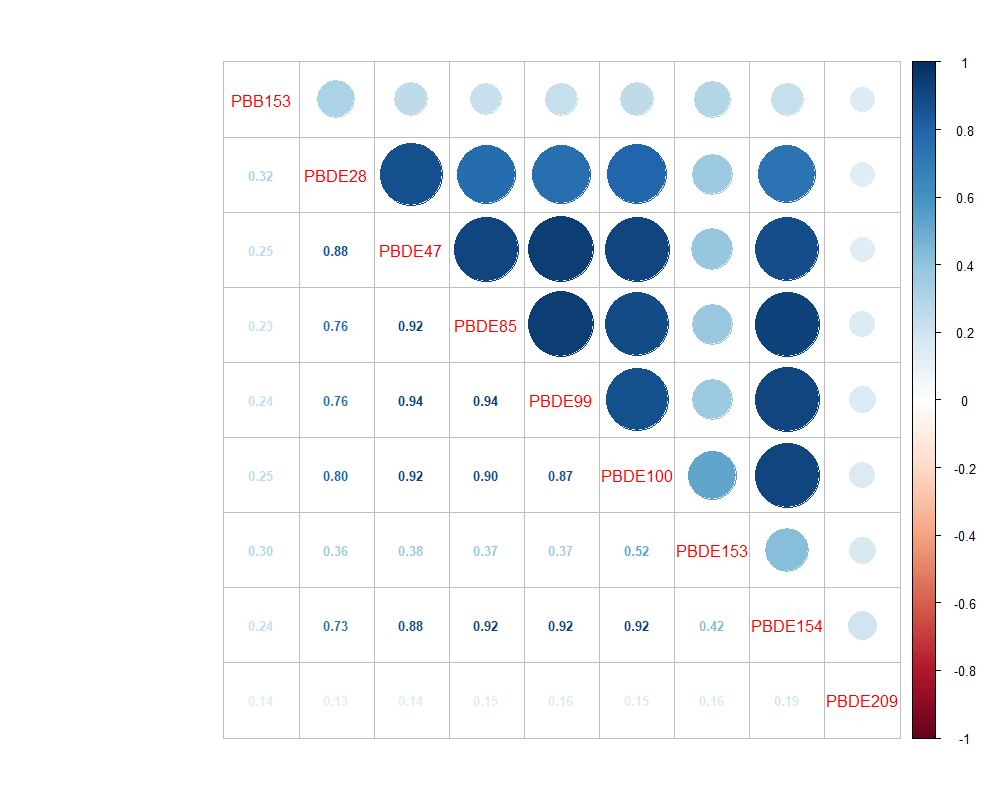


**Fig. S2. Pearson correlation coefficients between** **serum BFRs.**

Figure S3


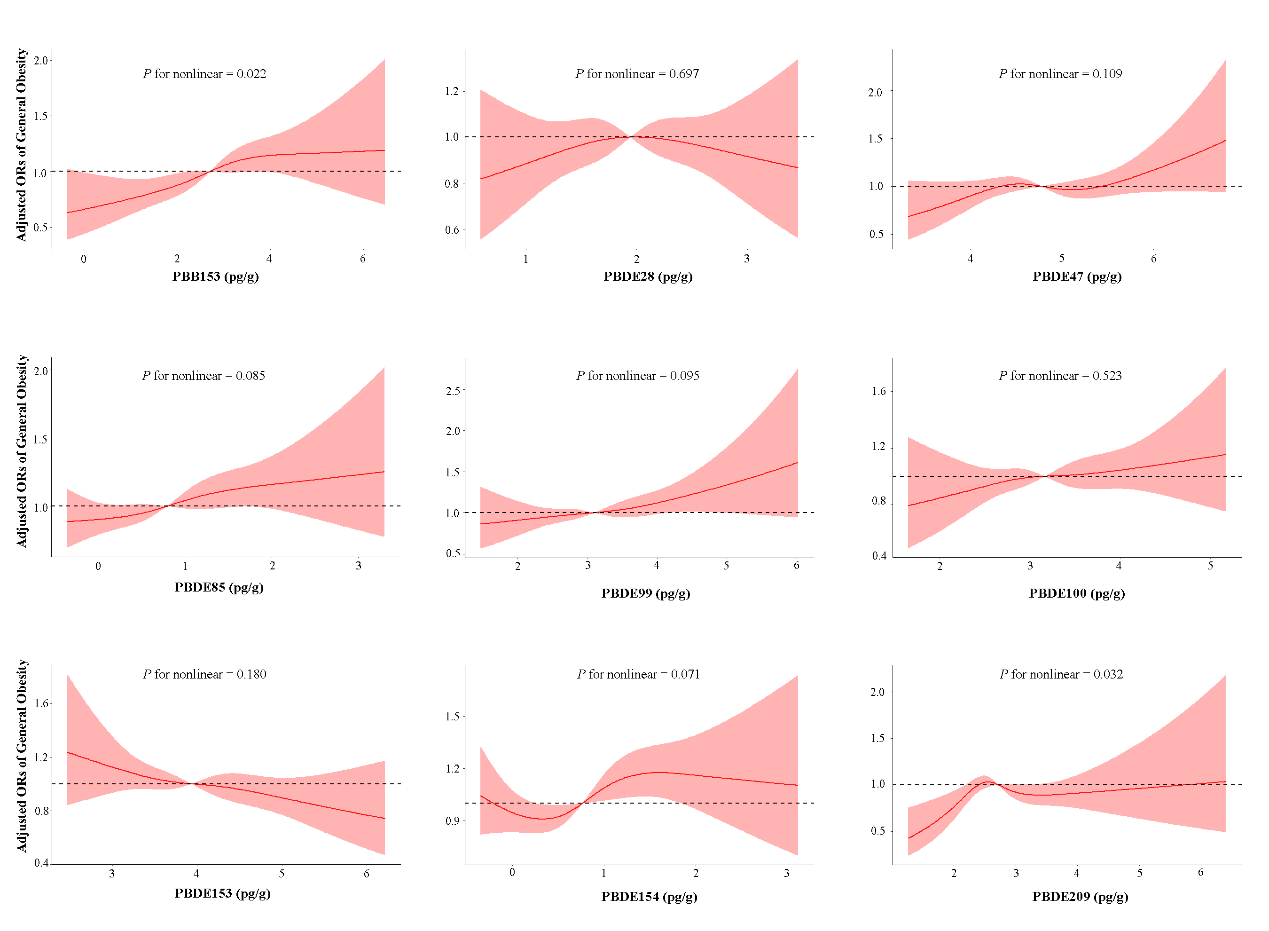


**Fig. S3. The continuous relationship of serum BFRs levels associated with general obesity based on RCS analysis.** The red solid lines represent the ORs, and red shadow range represent the 95 % CIs. The horizontal dashed line represents to the reference odds ratio of 1.0.

Figure S4


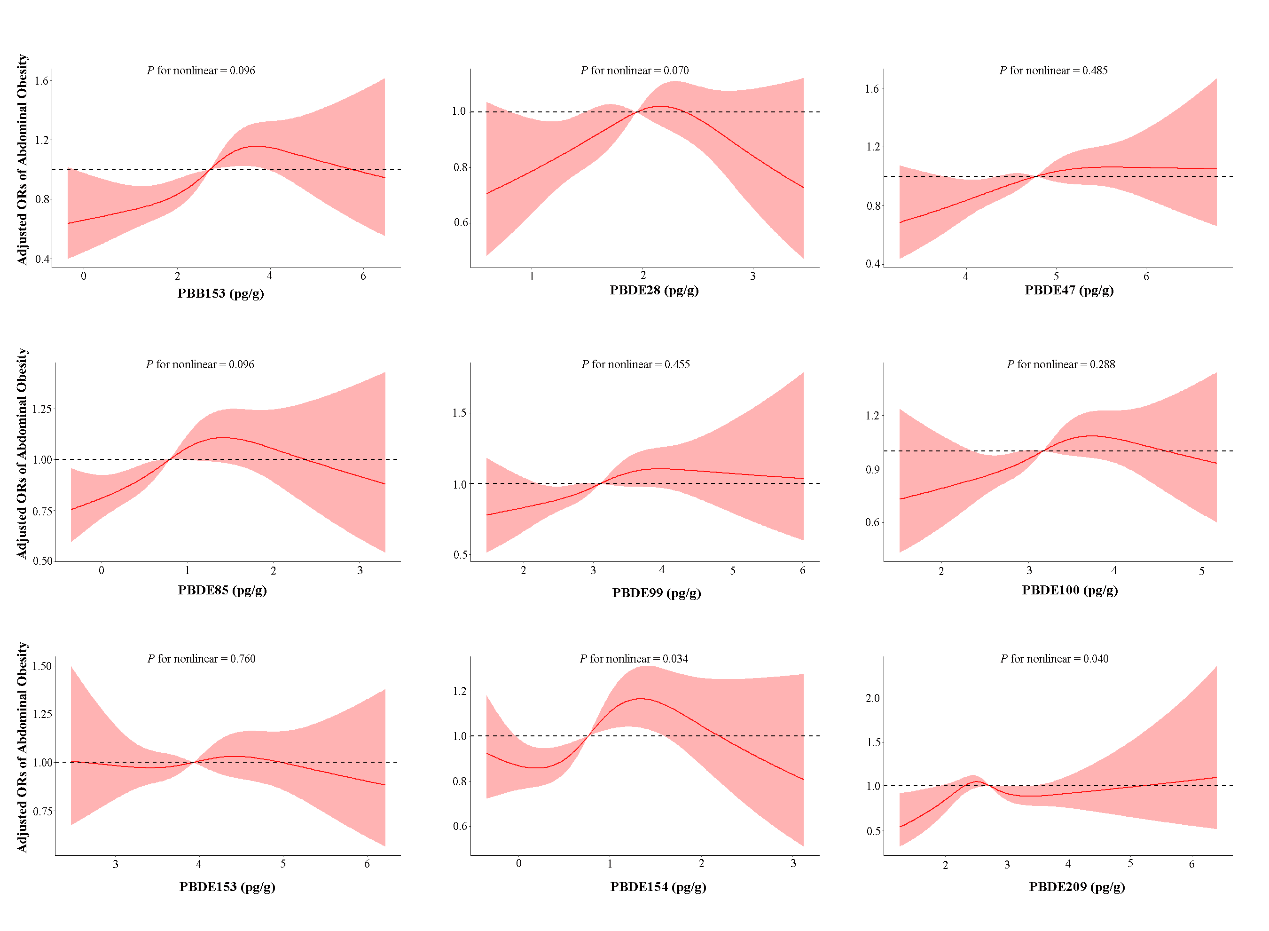


**Fig. S4. The continuous relationship of serum BFRs levels associated with abdominal obesity based on RCS analysis.** The red solid lines represent the ORs, and red shadow range represent the 95 % CIs. The horizontal dashed line represents to the reference odds ratio of 1.0.

Figure S5


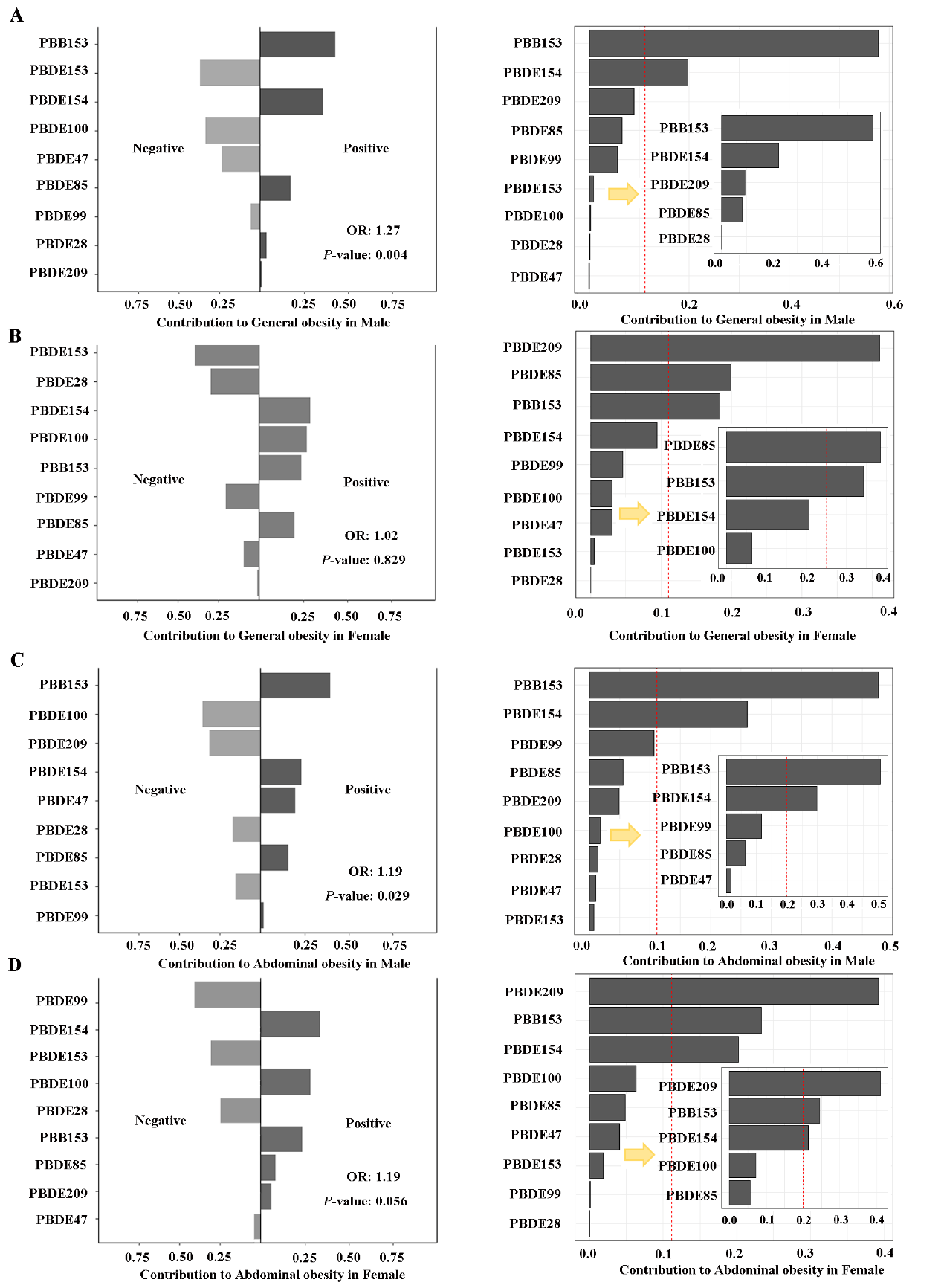


**Fig. S5.** **Association between In-transformed serum BFRs mixture and the risk of general obesity and abdominal obesity in male and female participants, as assessed via quantile-based g-computation (first step) and weighted quantile sum regression (second step after exclusion of exposure factors with negative weights).** (A) Association between In-transformed serum BFRs mixture and the risk of general obesity in male, (B) Association between In-transformed serum BFRs mixture and the risk of general obesity in female, (C) Association between In-transformed serum BFRs mixture and the risk of abdominal obesity in male, (D) Association between In-transformed serum BFRs mixture and the risk of abdominal obesity in female. All of the models are adjusted for demographic characteristics (age, race, educational levels, marital status and PIR) and lifestyle (cotinine levels and alcohol consumption).

Figure S6


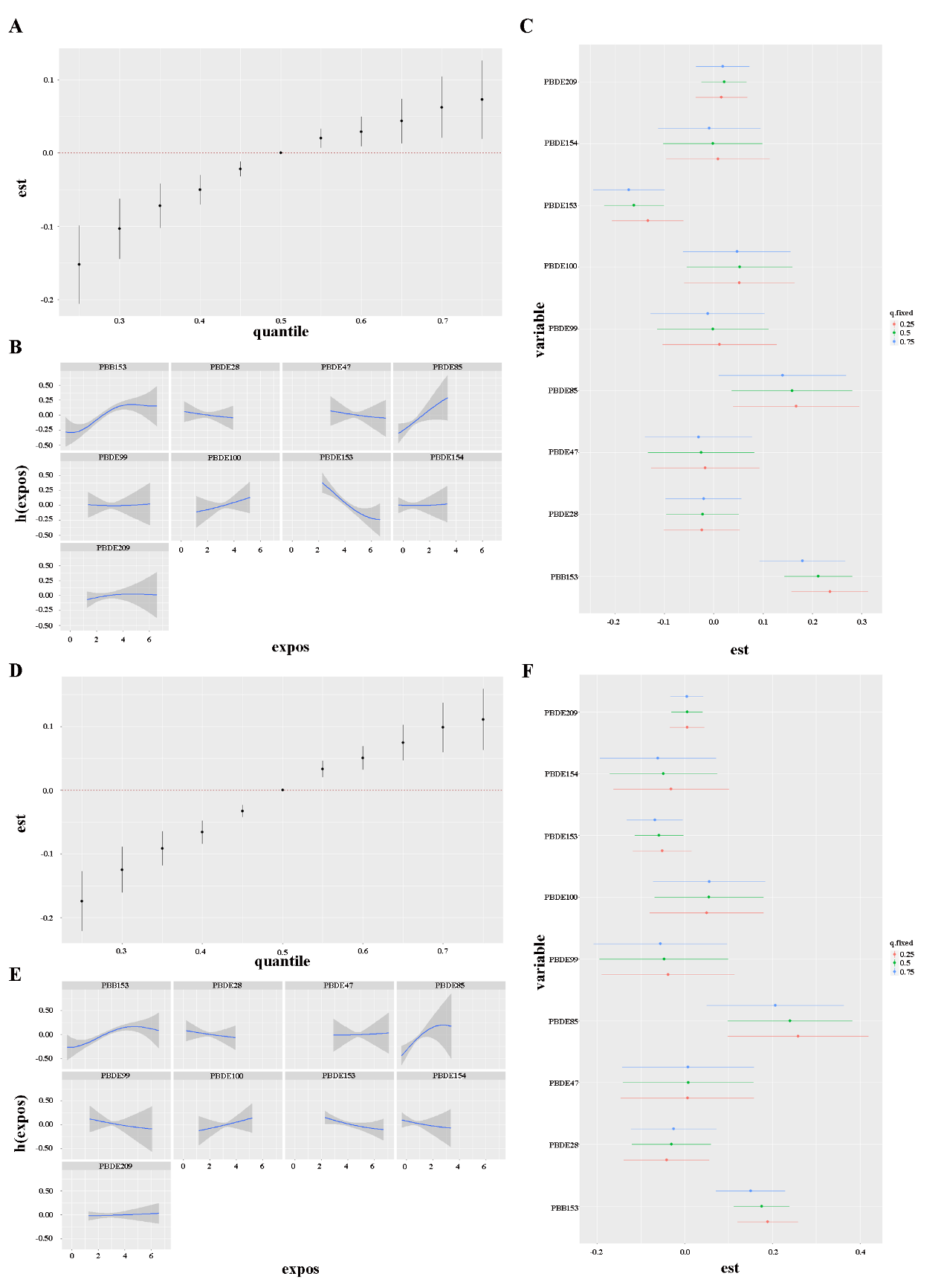


**Fig. S6. Association of obesity with BFRs estimated by Bayesian Kernel Machine Regression (BKMR) in participants.** All of the models are adjusted for demographic characteristics (gender, age, race, educational levels, marital status and PIR), lifestyle (cotinine levels and alcohol consumption) and self-reported of hypertension and diabetes conditions. (A), (D) Estimates and 95 % confidence intervals (95%CI) between quantiles of BFRs mixture and general obesity or abdominal obesity. Y-axis represents the estimated change in risk of the outcome when nine BFRs were set at particular percentiles (ranging from 25th to 75th) compared to the 50th percentile of each BFR. Dots indicate the estimate, and black vertical lines represent 95 % CIs. (B), (E) Univariate exposure-response functions (95%CI) in general obesity or abdominal obesity for single serum BFR when other eight serum BFRs fixed at the median. Y-axis represents the estimated difference in z-scores at a given level of serum BFR compared to its median level when the other eight serum BFRs were all at their median. (C), (F) The relationship of individual serum BFRs with general obesity or abdominal obesity when an individual BFR exposure was at its 75th percentile as compared to its 25th percentile, when all of the other BFRs were fixed at a specific exposure percentile (25th, 50th, or 75th, respectively).

Figure S7


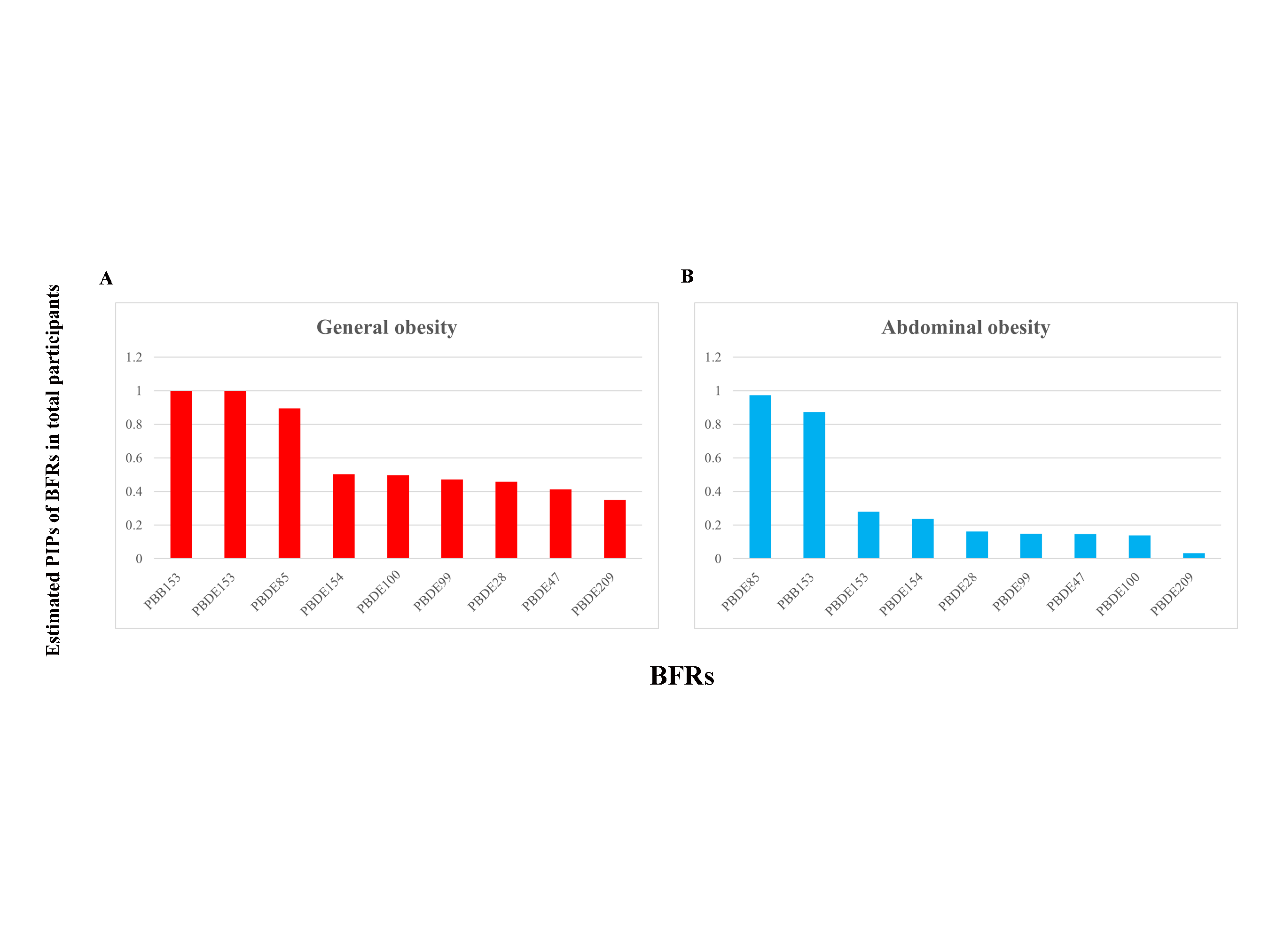


**Fig. S7. Posterior inclusion probabilities (PIPs) of each BFR for outcome, using the Bayesian kernel machine regression (BKMR) model.** (A) estimated PIPs of BFRs for general obesity, (B) estimated PIPs of BFRs for abdominal obesity. All of the models are adjusted for demographic characteristics (gender, age, race, educational levels, marital status and PIR), lifestyle (cotinine levels and alcohol consumption) and self-reported of hypertension and diabetes conditions.

**Table S1.** Detection rates of serum BFRs. NHANES 2009-2014 (N = 4110).

| Serum BFRs  (pg/g) | 2009-2010 | | | 2011-2012 | | | 2013-2014 | | |
| --- | --- | --- | --- | --- | --- | --- | --- | --- | --- |
|  | Pool number | ＜LLOD | Detection rate (%) | Pool number | ＜LLOD | Detection rate (%) | Pool number | ＜LLOD | Detection rate (%) |
| PBB153 | 301 | 39 | 87.04 | 251 | 15 | 94.02 | 284 | 22 | 92.25 |
| PBDE28 | 301 | 7 | 97.67 | 251 | 11 | 95.62 | 284 | 0 | 100.00 |
| PBDE47 | 301 | 0 | 100.00 | 251 | 0 | 100.00 | 284 | 0 | 100.00 |
| PBDE85 | 301 | 34 | 88.70 | 251 | 29 | 88.45 | 284 | 35 | 87.68 |
| PBDE99 | 301 | 0 | 100.00 | 251 | 0 | 100.00 | 284 | 00 | 100.00 |
| PBDE100 | 301 | 0 | 100.00 | 251 | 0 | 100.00 | 284 | 0 | 100.00 |
| PBDE153 | 301 | 0 | 100.00 | 251 | 0 | 100.00 | 284 | 0 | 100.00 |
| PBDE154 | 301 | 61 | 79.73 | 251 | 25 | 90.04 | 284 | 37 | 86.97 |
| PBDE209 | 301 | 12 | 96.01 | 251 | 4 | 98.41 | 284 | 6 | 97.89 |

Note：PBB153: 2,2´,4,4´,5,5´-Hexabromobiphenyl; PBDE28: 2,4,4´-Tribromodiphenyl ether; PBDE47: 2,2´,4,4´-Tetrabromodiphenyl ether; PBDE85: 2,2´,3,4,4´-Tentabromodiphenyl ether; PBDE99: 2,2´,4,4´,5-Pentabromodiphenyl ether; PBDE100: 2,2´,4,4´,6-Pentabromodiphenyl ether; PBDE153: 2,2´,4,4´,5,5´-Hexabromodiphenyl ether; PBDE154: 2,2´,4,4´,5,6´-Hexabromodiphenyl ether; PBDE209: Decabromodiphenyl ether. LLOD: lower limit of detection.

**Table S2.** Population characteristics by general obesity and sex in adults, NHANES 2009 - 2014.

| **catalogs** | **Males** | |  | **Females** | |  | **Total** |
| --- | --- | --- | --- | --- | --- | --- | --- |
|  | **Non**-**General obesity** | **General obesity** | ***P*-value** | **Non-General obesity** | **General obesity** | ***P*-value** |  |
| **Number of subjects (%) ^a^** | 1334 (65.3) | 708 (34.7) |  | 1236 (59.8) | 832 (40.2) |  | 4110 |
| **Age (%) ^a^** |  |  | 0.057 |  |  | 0.003 |  |
| 20-40 years | 473 (66.7) | 236 (33.3) |  | 458 (64.9) | 248 (35.1) |  | 1415 (34.4) |
| 40-60 years | 415 (61.8) | 257 (38.2) |  | 387 (56.5) | 298 (43.5) |  | 1357 (33.0) |
| ≥60 years | 446 (57.5) | 215 (32.5) |  | 391 (57.8) | 286 (42.2) |  | 1338 (32.6) |
| **Race (%)** **^a^** |  |  | < 0.001 |  |  | < 0.001 |  |
| Mexican American | 160 (56.9) | 121 (43.1) |  | 158 (55.4) | 127 (44.6) |  | 566 (13.8) |
| Other Hispanic | 123 (61.2) | 78 (38.8) |  | 127 (59.1) | 88 (40.9) |  | 416 (10.1) |
| Non-Hispanic White | 586 (65.8) | 305 (34.2) |  | 587 (63.2) | 342 (36.8) |  | 1820 (44.3) |
| Non-Hispanic Black | 275 (62.6) | 164 (37.4) |  | 169 (41.2) | 241 (58.8) |  | 849 (20.7) |
| Other race | 190 (82.6) | 40 (17.4) |  | 195 (85.2) | 34 (14.8) |  | 459 (11.2) |
| **Educational level (%) ^a^** |  |  | 0.119 |  |  | < 0.001 |  |
| Below high school | 301 (63.8) | 171 (36.2) |  | 249 (53.0) | 221 (47.0) |  | 942 (22.9) |
| High school | 311 (62.4) | 187 (37.6) |  | 245 (57.1) | 184 (42.9) |  | 927 (22.6) |
| Above high school | 722 (67.4) | 350 (32.6) |  | 742 (63.5) | 427 (36.5) |  | 2241 (54.5) |
| **Marital status (%) ^a^** |  |  | 0.128 |  |  | 0.011 |  |
| Married/living with partner | 835 (64.1) | 468 (35.9) |  | 709 (62.3) | 429 (37.7) |  | 2441 (59.4) |
| Widowed/divorced/separated/  never married | 499 (67.5) | 240 (32.5) |  | 527 (56.7) | 403 (43.3) |  | 1669 (40.6) |
| **Poverty income ratio (%) ^a^** |  |  | 0.294 |  |  | < 0.001 |  |
| ≤1.3 | 418 (66.7) | 209 (33.3) |  | 395 (55.1) | 322 (44.9) |  | 1344 (32.7) |
| 1.3–3.5 | 477 (63.2) | 278 (36.8) |  | 432 (58.6) | 305 (41.4) |  | 1492 (36.3) |
| >3.5 | 439 (66.5) | 221 (33.5) |  | 409 (66.6) | 205 (33.4) |  | 1274 (31.0) |
| **Body mass index (kg/m^2^),**  **(mean (SD))^b^** | 25.38 (2.88) | 35.13 (5.30) | < 0.001 | 24.56 (3.17) | 36.50 (6.11) | < 0.001 | 29.06 (6.79) |
| **Waist Circumference (cm), (mean (SD))^b^** | 92.95 (9.93) | 116.96 (13.24) | < 0.001 | 87.25 (9.39) | 112.44 (12.96) | < 0.001 | 99.32 (16.37) |
| **Cotinine level (%) ^a^** |  |  | 0.939 |  |  | 0.126 |  |
| Below LLOD | 303 (65.6) | 159 (34.4) |  | 432 (62.2) | 263 (37.8) |  | 1157 (28.2) |
| Above LLOD | 1031 (65.3) | 549 (34.7) |  | 804 (58.6) | 569 (41.4) |  | 2953 (71.8) |
| **Alcohol consumption (%) ^a^** |  |  | 0.864 |  |  | 0.139 |  |
| 12 drinks or fewer | 202 (64.7) | 110 (35.3) |  | 442 (57.6) | 325 (42.4) |  | 1079 (26.3) |
| More than 12 drinks | 1132 (65.4) | 598 (34.6) |  | 794 (61.0) | 507 (39.0) |  | 3031 (73.7) |
| **Hypertension (%) ^a^** | 399 (54.4) | 335 (45.6) | < 0.001 | 342 (45.7) | 406 (54.3) | < 0.001 | 1482 (36.1) |
| **Diabetes (%) ^a^** | 120 (47.1) | 135 (52.9) | < 0.001 | 88 (33.3) | 176 (66.7) | < 0.001 | 519 (12.6) |

^a^ Number of participants and percentage. Chi-square test was used to compare the differences of categorical variables between participants with and without general obesity.

^b^ Mean value and standard deviation (SD). Student’s t-test was used to compare the differences of continuous variables between participants with and without general obesity.

Note: Individuals with a body mass index (BMI) of 30 kg/m^2^ or more are classified as general obesity. Abdominal obesity is defined as a waist circumference of 102 centimeters or more for men and 88 centimeters or more for women.

**Table S3.** Population characteristics by abdominal obesity and sex in adults, NHANES 2009 - 2014.

| **catalogs** | **Males** | |  | **Females** | |  | **Total** |
| --- | --- | --- | --- | --- | --- | --- | --- |
| **Abdominal obesity** | **Non-Abdominal obesity** | **Abdominal obesity** | ***P*-value** | **Non-Abdominal obesity** | **Abdominal obesity** | ***P*-value** |  |
| **Number of subjects (%) ^a^** | 1138 (59.7) | 904 (40.3) |  | 639 (30.9) | 1429 (69.1) |  | 4110 |
| **Age (%) ^a^** |  |  | < 0.001 |  |  | < 0.001 |  |
| 20-40 years | 476 (67.1) | 233 (32.9) |  | 311 (44.1) | 395 (55.9) |  | 1415 (34.4) |
| 40-60 years | 360 (53.6) | 312 (46.4) |  | 186 (27.2) | 499 (72.8) |  | 1357 (33.0) |
| ≥60 years | 302 (45.7) | 359 (54.3) |  | 142 (21.0) | 535 (79.0) |  | 1338 (32.6) |
| **Race (%) ^a^** |  |  | < 0.001 |  |  | < 0.001 |  |
| Mexican American | 148 (52.7) | 133 (47.3) |  | 63 (22.1) | 222 (77.9) |  | 566 (13.8) |
| Other Hispanic | 121 (60.2) | 80 (39.8) |  | 59 (27.4) | 156 (72.6) |  | 416 (10.1) |
| Non-Hispanic White | 436 (48.9) | 455 (51.1) |  | 308 (33.2) | 621 (66.8) |  | 1820 (44.3) |
| Non-Hispanic Black | 253 (57.6) | 186 (42.4) |  | 78 (19.0) | 332 (81.0) |  | 849 (20.7) |
| Other race | 180 (78.3) | 50 (21.7) |  | 131 (57.2) | 98 (42.8) |  | 459 (11.2) |
| **Educational level (%) ^a^** |  |  | 0.293 |  |  | < 0.001 |  |
| Below high school | 259 (54.9) | 213 (45.1) |  | 93 (19.8) | 377 (80.2) |  | 942 (22.9) |
| High school | 265 (53.2) | 233 (46.8) |  | 122 (28.4) | 307 (71.6) |  | 927 (22.6) |
| Above high school | 614 (57.3) | 458 (42.7) |  | 424 (36.3) | 745 (63.7) |  | 2241 (54.5) |
| **Marital status (%) ^a^** |  |  | 0.022 |  |  | 0.256 |  |
| Married/living with partner | 701 (53.8) | 602 (46.2) |  | 364 (32.0) | 774 (68.0) |  | 2441 (59.4) |
| Widowed/divorced/separated/  never married | 437 (59.1) | 302 (40.9) |  | 275 (29.6) | 655 (70.4) |  | 1669 (40.6) |
| **Poverty income ratio (%) ^a^** |  |  | 0.769 |  |  | < 0.001 |  |
| ≤1.3 | 356 (56.8) | 271 (43.2) |  | 191 (26.6) | 526 (73.4) |  | 1344 (32.7) |
| 1.3–3.5 | 414 (54.8) | 341 (45.2) |  | 218 (29.6) | 519 (70.4) |  | 1492 (36.3) |
| >3.5 | 368 (55.8) | 292 (44.2) |  | 230 (37.5) | 384 (62.5) |  | 1274 (31.0) |
| **Body mass index (kg/m^2^),**  **(mean (SD)) ^b^** | 25.07  (3.06) | 33.40  (5.70) | < 0.001 | 22.51  (2.64) | 32.43  (6.81) | < 0.001 | 29.06  (6.79) |
| **Waist Circumference (cm), (mean (SD)) ^b^** | 90.15  (8.03) | 115.28 (12.02) | < 0.001 | 79.88  (5.74) | 105.21  (13.49) | < 0.001 | 99.32  (16.37) |
| **Cotinine level (%) ^a^** |  |  | 0.458 |  |  | 0.279 |  |
| Below LLOD | 250 (54.1) | 212 (45.9) |  | 226 (32.5) | 469 (67.5) |  | 1157 (28.2) |
| Above LLOD | 888 (56.2) | 692 (43.8) |  | 413 (30.1) | 960 (69.9) |  | 2953 (71.8) |
| **Alcohol consumption (%) ^a^** |  |  | 0.841 |  |  | 0.021 |  |
| 12 drinks or fewer | 176 (56.4) | 136 (43.6) |  | 213 (27.8) | 554 (72.2) |  | 1079 (26.3) |
| More than 12 drinks | 962 (55.6) | 768 (44.4) |  | 426 (32.7) | 875 (67.3) |  | 3031 (73.7) |
| **Hypertension (%) ^a^** | 287 (39.1) | 447 (60.9) | < 0.001 | 129 (17.2) | 619 (82.8) | < 0.001 | 1482 (36.1) |
| **Diabetes (%) ^a^** | 80 (31.4) | 175 (68.6) | < 0.001 | 25 (9.5) | 239 (90.5) | < 0.001 | 519 (12.6) |

^a^ Number of participants and percentage. Chi-square test was used to compare the differences of categorical variables between participants with and without abdominal obesity.

^b^ Mean value and standard deviation (SD). Student’s t-test was used to compare the differences of continuous variables between participants with and without abdominal obesity.

Note: Individuals with a body mass index (BMI) of 30 kg/m^2^ or more are classified as general obesity. Abdominal obesity is defined as a waist circumference of 102 centimeters or more for men and 88 centimeters or more for women.

**Table S4.** Difference in arithmetic mean of serum BFRs between male and female. NHANES 2009-2014 (N = 4110).

| Serum BFRs (pg/g) | Male | Female | *p* |
| --- | --- | --- | --- |
| PBB153 | 34.345 | 20.299 | < 0.001 |
| PBDE28 | 8.458 | 7.600 | < 0.001 |
| PBDE47 | 165.840 | 133.769 | < 0.001 |
| PBDE85 | 3.458 | 2.749 | < 0.001 |
| PBDE99 | 36.735 | 27.379 | < 0.001 |
| PBDE100 | 33.665 | 27.934 | < 0.001 |
| PBDE153 | 82.619 | 59.617 | < 0.001 |
| PBDE154 | 3.279 | 2.520 | < 0.001 |
| PBDE209 | 23.488 | 17.758 | < 0.001 |

Note：PBB153: 2,2´,4,4´,5,5´-Hexabromobiphenyl; PBDE28: 2,4,4´-Tribromodiphenyl ether; PBDE47: 2,2´,4,4´-Tetrabromodiphenyl ether; PBDE85: 2,2´,3,4,4´-Tentabromodiphenyl ether; PBDE99: 2,2´,4,4´,5-Pentabromodiphenyl ether; PBDE100: 2,2´,4,4´,6-Pentabromodiphenyl ether; PBDE153: 2,2´,4,4´,5,5´-Hexabromodiphenyl ether; PBDE154: 2,2´,4,4´,5,6´-Hexabromodiphenyl ether; PBDE209: Decabromodiphenyl ether. ^a^ G-Mean (95%). ^b^ Median (25th, 75th percentiles).
